# Development and validation of a nomogram for differentiating immune checkpoint inhibitor-related pneumonitis from pneumonia in patients undergoing immunochemotherapy: a multicenter, real-world, retrospective study

**DOI:** 10.3389/fimmu.2025.1495450

**Published:** 2025-05-19

**Authors:** Linli Duan, Guanglu Liu, Zijie Huang, Rong Chen, Di Mo, Yuxiao Xia, Jiazhu Hu, Mengzhang He

**Affiliations:** ^1^ Department of Pulmonary and Critical Care Medicine, The Second Affiliated Hospital, Guangzhou Medical University, Guangzhou, China; ^2^ Algorithm Development Department 1, GRGBanking Equipment Company Ltd., Guangzhou, China; ^3^ Thoracic Surgery Department, Guangzhou Institute of Cancer Research, the Affiliated Cancer Hospital, Guangzhou Medical University, Guangzhou, China; ^4^ Department of Oncology, The Affiliated Panyu Central Hospital of Guangzhou Medical University, Guangzhou, China

**Keywords:** checkpoint inhibitor-related pneumonitis (CIP), immunochemotherapy, nomogram, differentiate, pneumonia

## Abstract

**Background:**

Immune Checkpoint Inhibitor-related Pneumonitis (CIP) exhibits high morbidity and mortality rates in the real world, often coexisting with pneumonia, particularly after immunochemotherapy. We aimed to develop and validate a non-invasive nomogram for differentiating CIP from pneumonia in patients undergoing immunochemotherapy.

**Methods:**

This study encompassed 237 patients from three hospitals. A multivariate logistic regression analysis was conducted to identify risk factors for CIP. Utilizing the random forest machine learning method, optimal development and validation cohort allocation ratios (in a ratio of 8:2) were determined for the predictive model. The performance of the nomogram was evaluated using calibration, the area under the receiver operating characteristic curve (AUC), and decision curve analysis (DCA). Subsequently respiratory pathogens, management, and outcomes were compared between CIP and No CIP cases.

**Results:**

Among the 237 patients, 104 were diagnosed with CIP, and 133 were no CIP but pneumonia(No CIP). Smoking status, prior chronic obstructive pulmonary disease (COPD), ground glass opacities, non-specific interstitial pneumonitis, Neutrophil to Lymphocyte Ratio (NLR), pleural effusions, and Oxygen Partial Pressure (PaO_2_) emerged as non-invasive independent predictors of CIP. The nomogram exhibited good discrimination for both the development and validation cohorts, with AUC values of 0.817 (95% CI, 0.754–0.879) and 0.913 (95% CI, 0.826–0.999), respectively. The calibration curves demonstrated good fit for both the development and validation cohort, as evidenced by the Hosmer-Lemeshow tests (χ² = 3.939, *p* = 0.863 and χ² = 8.117, *p* = 0.422, respectively). DCA further highlighted their clinical utility. In CIP patients, the use of gamma globulin/albumin and glucocorticoids was significantly higher than in No CIP patients (39.4% vs 23.3%, *p* = 0.007; 79.8% vs 12.8%, *p* < 0.0001, respectively). The proportion of patients requiring mechanical ventilation was also significantly higher in the CIP compared to the No CIP group (21.2% vs 11.3%, *p* = 0.038).

**Conclusion:**

The nomogram offers a non-invasive approach to differentiate CIP from pneumonia associated with immunochemotherapy, potentially facilitating early intervention and informed treatment decisions.

## 1 Introduction

Immune checkpoint inhibitors (ICIs), such as anti-programmed cell death protein-1 (PD-1) and anti-programmed cell death ligand-1 (PD-L1) agents, have been approved for the treatment of various cancers, including non-small cell lung cancer (NSCLC), advanced melanoma, urothelial carcinoma, and hepatocellular carcinoma, among others (1). The advent of PD-1/PD-L1 has revolutionized the treatment of solid malignancies, offering hope to patients with advanced-stage tumors (2–6), particularly improving the prognosis of those with NSCLC (7, 8). ICIs have the potential to induce long-lasting remissions, with durable responses that can persist even after treatment discontinuation, leading to improved survival outcomes. Studies indicate that approximately 10-40% of patients with advanced cancer benefit from ICIs (9). These agents have demonstrated significant therapeutic efficacy. However, the activation of the immune system by ICIs can also lead to life-threatening immune-related adverse events (irAEs) (10, 11). IrAEs may necessitate treatment interruptions, dose reductions, or even the permanent discontinuation of ICIs.

Immune checkpoint inhibitor-related pneumonitis (CIP) is a life-threatening immune-related adverse event(irAE) that may induce respiratory failure (12). In clinical studies, the incidence rate of CIP is reported to be between 3-5% (13). However, an increasing number of real-world data suggest a significantly elevated incidence of CIP, reaching up to 13-30% (14–16), with 48% of cases classified as grade 3 or 4 and 5% as grade 5 pneumonitis (17). Another study also indicates that 41.8% of CIP patients suffer from severe CIP grades 3-5 (18). The severity of CIP is inversely related to the resolution of pneumonitis. The overall mortality rate due to adverse events (AEs) caused by PD-1/PD-L1 is 0.45%, with CIP being the most frequent cause of death, accounting for 28.0% of these fatalities (19). During treatment with PD-1 or PD-L1, the mortality rate due to pneumonitis is alarmingly high, reaching up to 35% (20). Furthermore, CIP also leads to an increased average length of hospital stay and medical costs, thereby amplifying the financial burden (21).

The high incidence and mortality rates of CIP are linked to the absence of specific clinical presentations and imaging characteristics in clinical settings. Previous studies have indicated that a history of pulmonary disease, smoking, radiotherapy, age, baseline proportion of CD4+ T lymphocytes and Absolute Eosinophil Count (AEC) are independent risk factors for the development of CIP (14, 22–24). However, these findings are primarily based on retrospective clinical studies from single centers with limited sample sizes. Moreover, patients with pulmonary infections were generally excluded from the CIP cohorts, whereas in real-world scenarios, CIP patients often present with concurrent pneumonitis, including special pathogens such as fungi, viruses, and tuberculosis, for which clinicians commonly employ anti-infective treatments. Some scholars categorized the clinical phenotypes of CIP into pure, induced, and mixed types, with the latter two accounting for up to 61.8% of cases. The induced and mixed types showed significantly higher rates of antibiotic usage compared to the pure type (71.4% vs 80.0% vs 23.8%, *p*=0.001), with the induced type having a high rate of antiviral use (85.7%), and the mixed type necessitating combined appropriate antibiotic therapy (18). The high proportion of CIP patients receiving antibiotics may correlate with the severity of CIP and the early suspicion of bacterial pneumonia.

Combining ICIs with chemotherapy is increasingly prevalent in clinical settings, yet it has been associated with a significant rise in pneumonitis rates, as evidenced in the KEYNOTE-407 trials, where both any-grade and severe (grades 3-5) pneumonitis were notably more frequent compared to ICIs monotherapy (25, 26). However, data on the real-world risks of CIP associated with this combined treatment are limited. Given that clinical trials often enroll patients with better health profiles, the risk of CIP in the real-world setting may significantly deviate from clinical trial results. Further research is urgently needed to clarify the risk of CIP with combined therapies.

CIP presents with non-specific clinical and radiographic findings, and patients often decline invasive diagnostic procedures such as bronchoscopy with biopsy. In the real world, CIP often overlaps with pneumonia, especially in patients treated with immunochemotherapy, making the differential diagnosis challenging for physicians. There is a paucity of research on the development of CIP risk prediction models for this patient cohort. Nomograms, which are based on core indicators, facilitate comprehensive patient evaluation. This study retrospectively reviews patients with new pulmonary lesions following immunochemotherapy at three centers. And we analyzed the clinical characteristics, pathogens, managements, and outcomes of CIP and No CIP.

Our aim is to develop and validate a machine learning-based model to predict the probability of CIP in these patients. The objective is to promptly identify high-risk CIP patients, enabling early and precise clinical decision-making.

## 2 Materials and methods

### 2.1 Study data

The retrospective study analyzes patients who developed pulmonary infections following treatment with immunochemotherapy at the Second Affiliated Hospital, the Cancer Hospital, and the Panyu Central Hospital of Guangzhou Medical University from January 1, 2018, to December 31, 2023. Initially, patients diagnosed with CIP were identified. Subsequently, a control group was selected, comprising patients who exhibited pneumonia during the same period but were not diagnosed with CIP. The inclusion criteria were as follows: 1) Age 18 years or older; 2) A confirmed diagnosis of cancer based on pathological and clinical findings; 3) Receipt of immunochemotherapy. Exclusion criteria included: 1) Progression of pulmonary tumor infiltration; 2) Radiation-induced lung disease; 3) Stage I-II cancer; 4) Lack of baseline radiological data. The study was approved by the Ethics Committee of the Second Affiliated Hospital of Guangzhou Medical University (No.2024-hg-ks-41).

### 2.2 Definition and diagnosis of CIP and pneumonia

CIP is defined as the focal or diffuse immunological inflammation of the lung parenchyma occurring after treatment with ICIs. Pneumonia is defined as the infection of the lung caused by bacteria, fungi, or viruses following immunochemotherapy.

Due to the lack of standardized diagnostic criteria for CIP, its clinical presentation is non-specific, and it is a diagnosis of exclusion. According to the 2019 NCCN guidelines (27), the diagnosis of CIP is based on computed tomography (CT) imaging and clinical signs, excluding disease progression, pulmonary infection, and radiation pneumonitis. In real-world settings, particularly when CIP and pneumonia coexist, diagnosis is challenging. Sometimes a diagnosis of CIP is considered after antibiotic treatment shows no significant improvement or only partial improvement in lung lesions, but there is a marked response to glucocorticoid.

In this study, the diagnosis of CIP was collaboratively determined by experienced radiologists, oncologists, and pulmonologists, following the 2019 NCCN guidelines (27) and by assessing clinical and radiological characteristics. Patients whose clinical and radiographic presentations were consistent with CIP, yet could not definitively rule out concurrent cardiopulmonary diseases such as volume overload or positive respiratory pathogen tests, were considered for the CIP group (14). Patients who developed new pneumonia, diagnosed by combining clinical manifestations, chest CT, and microbiological testing, but were not diagnosed with CIP after excluding tumor progression, were classified as No CIP group.

### 2.3 Study outcome and variables

The data were extracted from the electronic medical record system, meticulously documenting patient demographics, smoking history, laboratory test results, thoracic CT imaging, history of underlying diseases, tumor histological types and staging, sputum pathogen profiles, antibiotic, treatments, and outcomes. This compilation is specific to the index admission for emergent pulmonary lesions subsequent to oncologic therapy.

### 2.4 Statistical analysis

Group comparisons were made using t-tests, chi-square tests, or Mann-Whitney U tests, complemented by Fisher’s exact test when necessary. Normally distributed data were expressed as mean ± Standard Deviation(SD), while non-normal distributions were represented by medians and Interquartile Ranges(IQRs). Logistic regression identified independent risk factors for CIP, with univariate and multivariate analyses applied. The random forest algorithm optimized the distribution ratio for predictive model development and validation cohort. The nomogram’s performance was assessed using receiver operating characteristic(ROC)curve area under the curve(AUC), calibration curves, and Decision curve analysis curves(DCA). An AUC > 0.7 indicated good discrimination, and a Hosmer-Lemeshow *p*-value > 0.05 indicated model fit (28, 29). DCA evaluated clinical utility (30). Statistical analyses and graphics were conducted in R (version 4.4.1) and Prism(10.2)and *p*< 0.05 were considered statistically significant, and all tests were two-tailed.

## 3 Results

### 3.1 Patients characteristics

After the evaluation and review of medical records and imaging data by respiratory medicine, radiology, and oncology specialists, a total of 237 patients from three centers were included in this study, with 104 patients in the CIP group and 133 in the No CIP group. All patients in the No CIP group had pneumonia, while those in the CIP group with or without pneumonia. Demographic data, laboratory tests, and chest imaging characteristics of the CIP and No CIP groups are detailed in **Table 1**. The proportions of lung cancer patients in the CIP and No CIP groups were 63.5% and 53.4%, respectively, with the remaining patients having tumors in the digestive system, urinary system, head and neck, and hematological system, among others. Significant differences (*p* < 0.05) were observed between the two groups in terms of Sex, Smoking status, Prior Chronic Obstructive Pulmonary Disease(COPD), Myelosuppression following Chemotherapy, Ground glass opacities (GGO), non-specific interstitial pneumonitis (NSIP), pleural effusions, Absolute Lymphocyte Count (ALC), Neutrophil-to-Lymphocyte Ratio (NLR), Platelet-to-Lymphocyte Ratio (PLR), and Partial Pressure of Oxygen in Arterial Blood (PaO_2_).

**Table 1:** Demographic and clinical characteristics of patients in CIP and No CIP.

Variables	Whole population (n=237)	CIP (n=104)	No CIP (n=133)	P *value*
Sex, No. (%)				0.0458
Male	175 (73.8)	84 (80.8)	91 (68.4)	
Female	62 (26.2)	20 (19.2)	42 (31.6)	
Age(year) (median [IQR])	63.0 [57.0, 70.0]	63.0 [58.0, 69.0]	63.0 [57.0, 70.0]	0.689
BMI(kg/m2),No. (%)				0.8625
<18.5	68 (28.7)	31 (29.8)	37 (27.8)	
18.5-24.9	134 (56.5)	59 (56.7)	75 (56.4)	
>=25	35 (14.8)	14 (13.5)	21 (15.8)	
Smoking status, No. (%)				<0.0001
Ex-smoker/smoker	111 (46.9)	68 (65.4)	43 (32.3)	
Non-smoker	126 (53.2)	36 (34.6)	90 (67.7)	
Lung Cancer, No. (%)				0.1537
Yes	137 (57.8)	66 (63.5)	71 (53.4)	
No	100 (42.2)	38 (36.5)	62 (46.6)	
TNM stage, No. (%)				0.8916
III	39 (16.5)	18 (17.3)	21 (15.8)	
IV	198 (83.5)	86 (82.7)	112 (84.2)	
Prior COPD, No. (%)				<0.0001
Yes	69 (29.1)	54 (51.9)	15 (11.3)	
No	168 (70.9)	50 (48.1)	118 (88.7)	
Underlying lung disease (Excluding COPD),No. (%)				1
Yes	25 (10.6)	11 (10.6)	14 (10.5)	
No	212 (89.5)	93 (89.4)	119 (89.5)	
Hypertension, No. (%)				0.2289
Yes	58 (24.5)	21 (20.2)	37 (27.8)	
No	179 (75.5)	83 (79.8)	96 (72.2)	
Diabetes, No. (%)				0.2942
Yes	30 (12.7)	10 (9.6)	20 (15.1)	
No	207 (87.3)	94 (90.4)	113 (85.0)	
CIBMS, No. (%)				0.0305
Yes	43 (18.1)	12 (11.5)	31 (23.3)	
No	194 (81.9)	92 (88.5)	102 (76.7)	
patchy consolidation, No. (%)				0.2049
Yes	117 (49.4)	46 (44.2)	71 (53.4)	
No	120 (50.6)	58 (55.8)	62 (46.6)	
GGO, No. (%)				0.0056
Yes	18 (7.6)	14 (13.5)	4 (3.0)	
No	219 (92.4)	90 (86.5)	129 (97.0)	
NSIP, No. (%)				0.0039
Yes	30 (12.7)	21 (20.20)	9 (6.8)	
No	207 (87.3)	83 (79.8)	124 (93.2)	
PNOS, No. (%)				0.1273
Yes	63 (26.6)	22 (21.2)	41 (30.8)	
No	174 (73.4)	82 (78.9)	92 (69.2)	
Organizing/hypersensitivity pneumonia, No. (%)				0.4492
Yes	4 (1.7)	3 (2.9)	1 (0.8)	
No	233 (98.3)	101 (97.1)	132 (99.3)	
pleural effusions, No. (%)				0.0028
Yes	68 (28.7)	19 (18.3)	49 (36.8)	
No	169 (71.3)	85 (81.7)	84 (63.2)	
With targeted Therapy, No. (%)				0.4416
Yes	57 (24.1)	22 (21.2)	35 (26.3)	
No	180 (76.0)	82 (78.9)	98 (73.7)	
Prior radiotherapy, No. (%)				0.0723
Yes	52 (22.0)	29 (27.9)	23 (17.3)	
No	185 (78.1)	75 (72.1)	110 (82.7)	
With anti angiogenesis therapy, No. (%)				0.9242
Yes	37 (15.6)	17 (16.4)	20 (15.0)	
No	200 (84.4)	87 (83.7)	113 (85.0)	
WBC(×109/L) (median [IQR])	7.9 [5.1, 12.4]	7.3 [4.91 11.9]	8.4[5.5, 12.6]	0.1522
ANC(×109/L) (median [IQR])	6.4 [3.9, 10.3]	6.1 [3.7, 9.6]	6.4[4.0, 10.7]	0.4265
ALC(×109/L) (median [IQR])	0.8[0.5, 1.2]	0.6[0.4, 1.0]	0.9[0.6, 1.4]	<0.0001
HB(g/L) (mean (SD))	94.9(25.5)	97.5(24.0)	92.9 (26.5)	0.1609
PLT(×10^9/L) (median [IQR])	189.0[98.0, 290.0]	189.5[110.3, 304.3]	185.0 [93.0, 263.0]	0.282
NLR (median [IQR])	8.1 [4.1, 15.4]	9.9 [5.3, 17.4]	6.6 [3.5, 12.6]	0.0064
PLR (median [IQR])	236.8 [137.9, 392.2]	314.0 [183.3, 429.5]	203.8 [116.9, 308.1]	0.0002
PaO2 (median [IQR])	88.0 [76.3, 103.6]	83.20[70.9, 95.3]	93.9 [81.0, 112.0]	0.0001
ALT(U/L) (median [IQR])	21.0 [12.0,36.0]	21.0 [14.8, 37.1]	21.0 [11.0, 34.0]	0.0719
AST(U/L) (median [IQR])	30.0 [19.0, 50.0]	31.0 [20.9, 47.8]	30.0 [17.0, 53.0]	0.2121
TBIL(umol/L) (median [IQR])	11.4 [7.9, 16.2]	11.5 [7.7, 15.9]	11.4 [8.0, 17.8]	0.8359
DBIL(umol/L) (median [IQR])	2.90 [1.80, 5.80]	3.15 [2.00, 5.9]	2.80 [1.70, 5.80]	0.2166
ALB(g/L) (median [IQR])	31.1[27.2, 35.4]	30.1 [27.1, 34.6]	32.0 [27.5, 36.0]	0.2492
BUN (mmol/L) (median [IQR])	5.7 [4.1, 7.8]	5.9 [4.2, 8.3]	5.3 [4.0, 7.4]	0.1069
Cr(umol/L) (median [IQR])	80.0 [63.0, 99.4]	81.0 [63.9, 96.6]	78.6 [61.9, 100.9]	0.8106
CK(U/L) (median [IQR])	47.0 [26.0, 91.0]	50.5 [27.0, 107.0]	45.0 [24.0, 73.0]	0.1374
CKMB(U/L) (median [IQR])	12.0 [9.0, 20.0]	12.0 [9.0, 20.3]	12.0 [8.0, 19.0]	0.7017
LDH(U/L) (median [IQR])	258.0 [181.0, 373.0]	269.0[187.78, 370.25]	235.0 [168.0, 386.0]	0.3345
PCT(ng/ml) (median [IQR])	0.3 [0.1, 0.9]	0.3 [0.1, 1.1]	0.2 [0.1, 0.8]	0.3624
D-Dimer(mg/L FEU) (median [IQR])	2.1 [0.9, 4.4]	2.2 [1.1, 4.7]	2.1 [0.8, 4.1]	0.2313
proteinuria, No. (%)				0.421
Yes	43 (18.1)	16 (15.4)	27 (20.3)	
No	194 (81.9)	88 (84.6)	106 (79.7)	
Fungi in sputum, No. (%)				0.5328
Yes	58 (24.5)	28 (26.9)	30 (22.6)	
No	179 (75.5)	76 (73.1)	103 (77.4)	

BMI, Body Mass Index; TNM, tumor node metastasis; COPD, chronic obstructive pulmonary disease; GGO, ground glass opacities; NSIP, non-specific interstitial pneumonitis; PNOS: pneumonitis not otherwise specified; CIBMS:Chemotherapy-induced bone marrow suppression;WBC, White Blood Cell; ANC, Absolute Neutrophil Count; ALC, absolute lymphocyte count; HB, Hemoglobin; PLT, Platelet; NLR, Neutrophil to Lymphocyte Ratio; PLR, Platelet to Lymphocyte Ratio;PaO2,Oxygen Partial Pressure; ALT, Alanine Aminotransferase; AST, Aspartate Aminotransferase; TBIL, Total Bilirubin; DBIL, Direct Bilirubin; ALB, Albumin; BUN, Blood Urea Nitrogen; Cr, Creatinine; CK, Creatine Kinase; CKMB, Creatine Kinase Myocardial Band; LDH, Lactate Dehydrogenase; PCT, Procalcitonin; D-Dimer, Degradation Product of Cross-linked Fibrin.

### 3.2 Univariate and multivariate analysis of risk factors associated with CIP

In the univariate analysis, multiple non-invasive clinical parameters were associated with CIP. In the multivariate analysis, Smoking status, Prior COPD, GGO, NSIP, NLR, pleural effusions and PaO_2_ were independent predictors of CIP (**Table 2**). Specifically, the odds ratios (OR) and 95% confidence intervals [CI] for Smoking status, Prior COPD, and NLR were 2.913 (1.249-6.97), 5.975 (2.578-14.571), and 1.065 (1.029-1.106), respectively. The OR for GGO and NSIP were 9.189 (2.385-43.336) and 3.006 (1.15-8.353), respectively. In contrast, pleural effusions and PaO_2_ exhibited protective effects with OR of 0.452 (95% CI: 0.201-0.979) and 0.974 (95% CI: 0.959-0.988), respectively. All variables analyzed showed statistical significance with *p*< 0.05.

**Table 2:** Univariate and multivariate logistic analysis for risk factors of CIP.

Variables	Univariate analysis		Multivariate analysis	
	OR(95% CI)	P value	OR(95% CI)	P value
Sex(Male VS Female)	1.938(1.054-3.566)	0.033	0.511(0.205-1.239)	0.141
Smoking status(Yes vs. No)	3.953(2.296-6.807)	<0.001	2.913(1.249-6.97)	**0.014**
Prior COPD(Yes vs. No)	8.496(4.388-16.451)	<0.001	5.975(2.578-14.571)	**<0.001**
CIBMS(Yes vs. No)	0.429(0.208-0.885)	0.022	0.41(0.158-1.015)	0.059
GGO(Yes vs. No)	5.017(1.599-15.739)	0.006	9.189(2.385-43.336)	**0.002**
NSIP(Yes vs. No)	3.486(1.522-7.985)	0.003	3.006(1.15-8.353)	**0.028**
pleural effusions(Yes vs. No)	0.383(0.208-0.705)	0.002	0.452(0.201-0.979)	**0.048**
ALC(×109/L)	0.952(0.85-1.066)	0.394	0.984(0.837-1.085)	0.777
NLR	1.038(1.01-1.066)	0.006	1.065(1.029-1.106)	**0.001**
PLR	1(1-1.001)	0.403	1(0.999-1)	0.36
PaO2	0.979(0.967-0.991)	<0.001	0.974(0.959-0.988)	**<0.001**

COPD, chronic obstructive pulmonary disease; GGO, ground glass opacities; NSIP, non-specific interstitial pneumonitis; CIBMS, Chemotherapy-induced bone marrow suppression; ALC, absolute lymphocyte count; NLR, Neutrophil to Lymphocyte Ratio; PLR, Platelet to Lymphocyte Ratio; PaO_2_, Oxygen Partial Pressure.

Bold indicates p<0.05.

### 3.3 Developing and validating the non-invasive risk assessment nomogram

**Figure 1:**
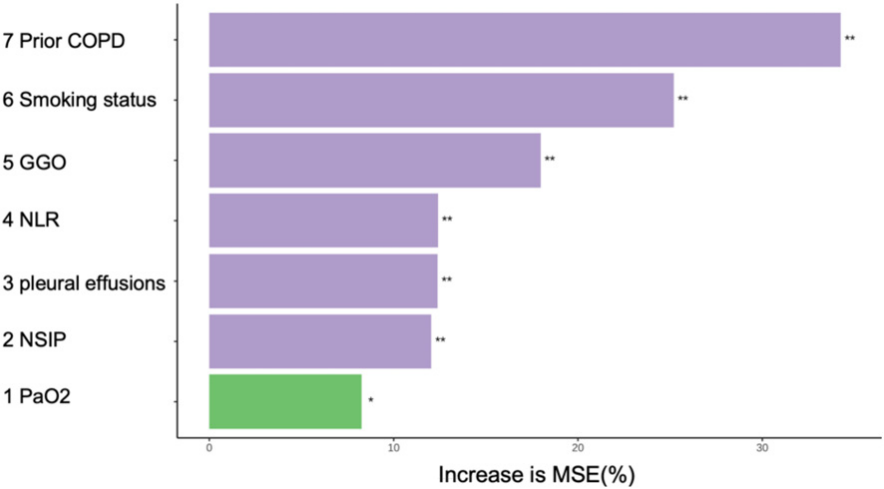
In random forests, the degree of influence of predictor variables on the model’s MSE. *p<0.05;**p<0.01. MSE, Mean Squared Error. COPD, chronic obstructive pulmonary disease; NLR, Neutrophil to Lymphocyte Ratio; GGO, ground glass opacities; NSIP, non-specific interstitial pneumonitis; PaO_2_, Oxygen Partial Pressure.

Before model construction, the dataset was split into development and validation cohort with an optimal 8:2 ratio identified by Random Forest, minimizing subjective bias. This ratio enhanced model performance, and the seven predictors showed no significant differences between cohorts (**Table 3**). the degree of influence of predictor variables on the model’s Mean Squared Error (MSE) is shown in **Figure 1**.

**Table 3:** Independent predictive factors in development and validation cohorts.

Variables	Overall population(n=237)	Development cohort (n=190)	Validation cohort(n=47)	p
Smoking status, No. (%)				0.6273
Ex-smoker/smoker	111 (46.84)	87 (45.79)	24 (51.06)	
Non-smoker	126 (53.16)	103 (54.21)	23 (48.94)	
Prior COPD, No. (%)				1
Yes	69 (29.11)	55 (28.95)	14 (29.79)	
No	168 (70.89)	135 (71.05)	33 (70.21)	
GGO, No. (%)	18 (7.59)	15 (7.89)	3 (6.38)	0.9659
Yes				
No	219 (92.41)	175 (92.11)	44 (93.62)	
NSIP, No. (%)				0.4776
Yes	30 (12.66)	26 (13.68)	4 (8.51)	
No	207 (87.34)	164 (86.32)	43 (91.49)	
pleural effusions, No. (%)				0.9958
Yes	68 (28.69)	54 (28.42)	14 (29.79)	
No	169 (71.31)	136 (71.58)	33 (70.21)	
NLR (median [IQR])	8.09 [4.14, 15.42]	8.73 [4.37, 15.65]	6.213 [3.410, 11.488]	0.0996
PaO2 (median [IQR])	88.00 [76.30, 103.60]	89.00 [76.95, 103.82]	86.10 [69.95, 103.00]	0.4123

COPD, chronic obstructive pulmonary disease; GGO, ground glass opacities; NSIP, non-specific interstitial pneumonitis; NLR, Neutrophil to Lymphocyte Ratio; PaO_2_, Oxygen Partial Pressure.

**Figure 2:**
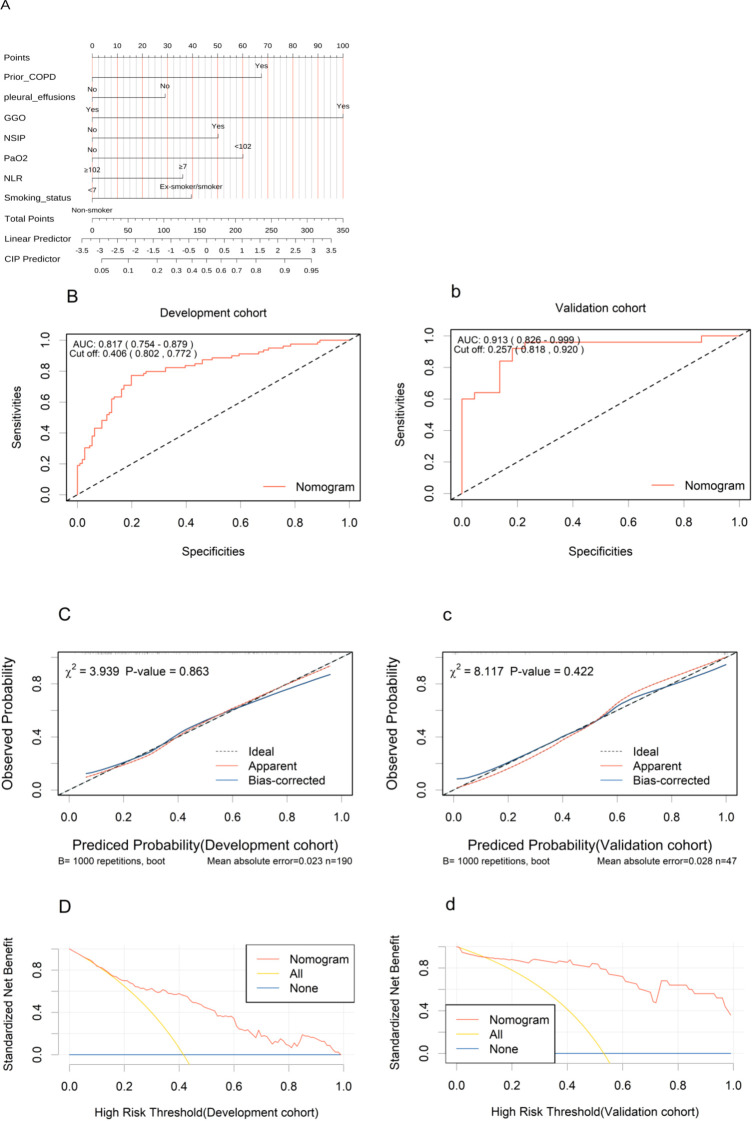
Development and validation of the nomogram. **(A)** The nomogram uses seven variables, assigning points for each. Add up the points to get a total score, which subsequently serves to estimate the predicted probability of CIP. (**B**, b), ROC curves in the development **(B)** and validation (b) cohorts. (**C**, c), Calibration curves in the nomogram in the development **(C)** and validation (c) cohorts. (**D**, d), Decision curve analysis curves for the nomogram model in the development **(D)** and validation (d) cohorts. ROC, receiver operating characteristic; AUC, area under the curve; COPD, chronic obstructive pulmonary disease; NLR, Neutrophil to Lymphocyte Ratio; GGO, ground glass opacities; NSIP, non-specific interstitial pneumonitis; PaO2, Oxygen Partial Pressure.

These seven independent predictors were used to construct a non-invasive clinical nomogram for predicting the risk of CIP (**Figure 2A**). We calculated the optimal cutoff scores for these independent predictive variables based on the Youden Index. The score for each variable is calculated based on the nomogram, and the total score corresponds to the predicted risk, which represents the risk of CIP. Clinical Application: A 60-year-old smoker with COPD (score=40), presenting with pleural effusions (score=0), GGO on chest CT (score=100), NSIP (score=50), PaO₂ =80 mmHg (score=60), and NLR=15 (score=36), accumulates a total score of 286. This corresponds to a CIP probability of approximately 90%.

The AUC values under the ROC curves for the development and validation cohorts were 0.817 (95% CI, 0.754–0.879; **Figure 2B**) and 0.913 (95% CI, 0.826–0.999; **Figure 2**b), respectively, indicating excellent discriminative power of the nomogram. The calibration curves demonstrated good fits for both the development and validation cohorts, with Hosmer-Lemeshow tests(χ² = 3.939, *p* = 0.863 and χ² = 8.117, *p* = 0.422, respectively) (**Figures 2C**, c). This indicated no significant difference between the predicted and observed probabilities. The DCA curve further confirmed their clinical utility, showing that in the development cohorts (**Figures 2D**), the nomogram provided greater net benefit compared to the “treat-all” and “treat-none” schemes. The DCA curve for the validation cohorts (**Figures 2**d), showed that if the threshold probability is > 10%, using the nomogram in the current study to predict CIP adds more benefit than the “treat-all” and “treat-none” schemes. These results indicate that the nomogram model can accurately predict the risk of CIP.

### 3.4 The pathogens, managements, and outcomes in CIP and no CIP

**Figure 3:**
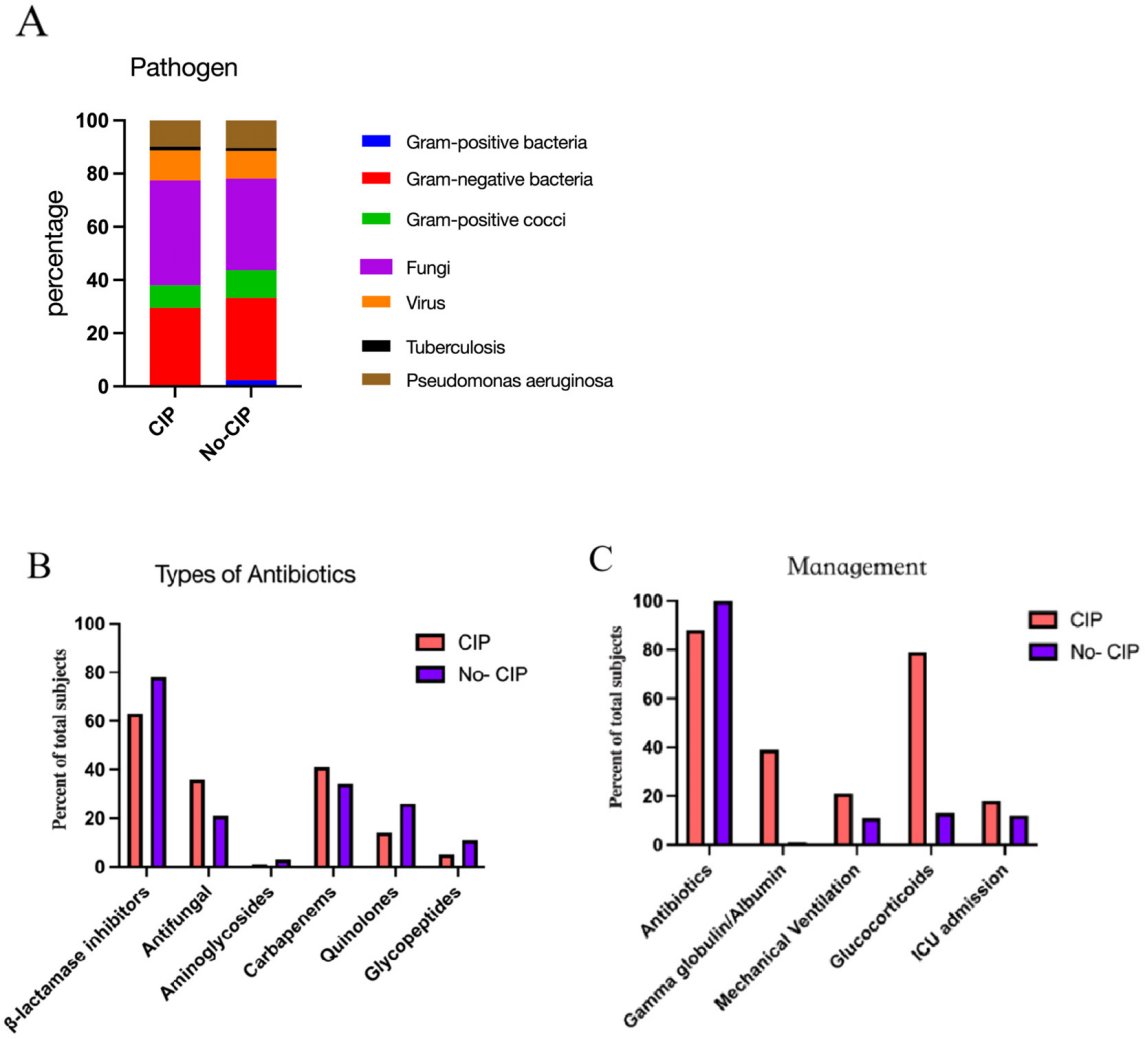
The pathogens**(A)**, Types of antibiotics **(B)**, and Managements **(C)** in CIP and No CIP.CIP, immune checkpoint inhibitor-related pneumonitis.

**Figure 4:**
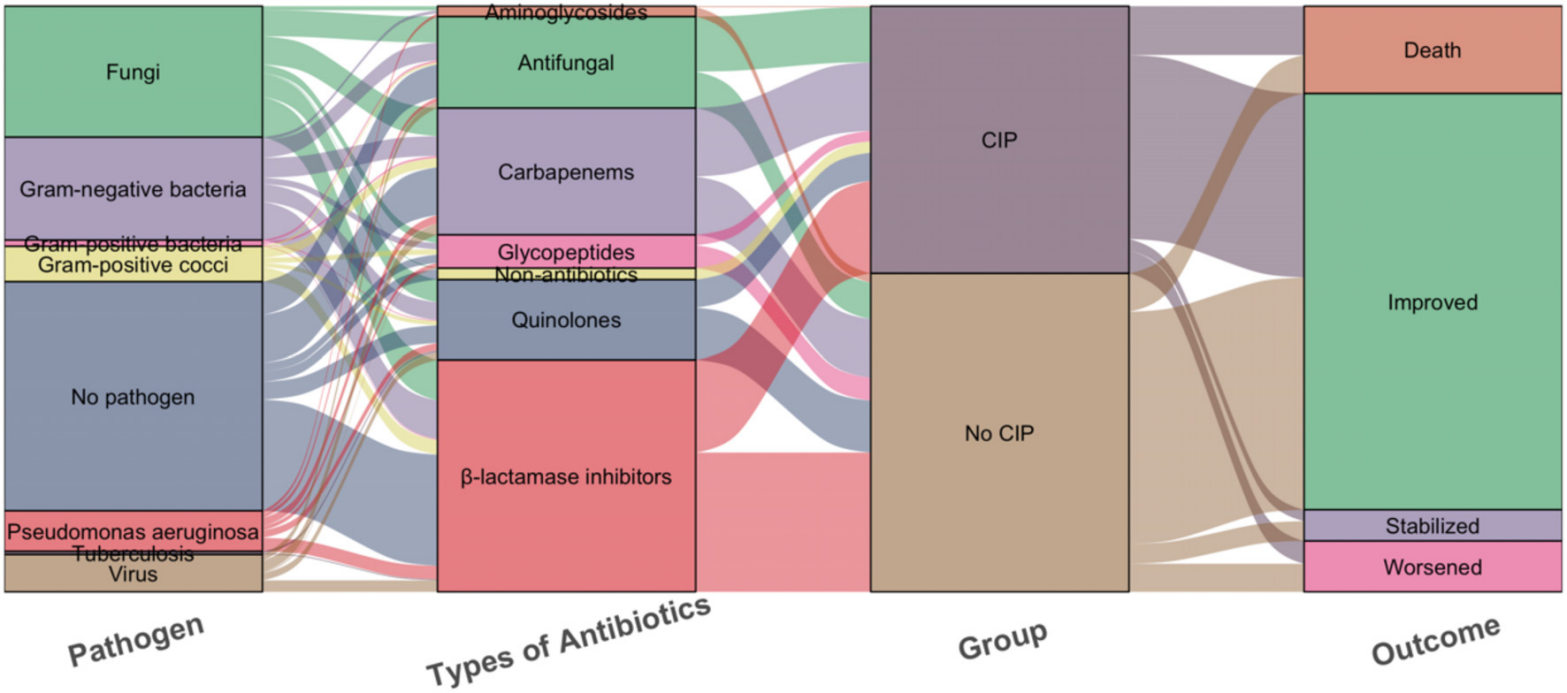
Sankey diagram of the pathogens, Types of antibiotics and outcomes with the CIP and No CIP. CIP, immune checkpoint inhibitor-related pneumonitis.

In pathogen detection, the methods included traditional sputum culture, Targeted Next-Generation Sequencing (tNGS) and Metagenomic Next-Generation Sequencing (mNGS), we identified pathogens in 33 patients with CIP and 50 with No CIP. Among these, NGS results were available for 22 cases. Regrettably, no significant difference in pathogen detection was observed between subgroups, with gram-negative bacteria and fungi being the most common. All No CIP patients received antibiotics, while 89.4% of CIP patients did. In CIP patients, gamma globulin/albumin and glucocorticoids usage was significantly higher than in No CIP (39.4% vs 23.3%, p=0.007; 79.8% vs 12.8%, *p*<0.0001). Mechanical ventilation rates were 21.2% in CIP vs 11.3% in No CIP (*p*=0.038). Antimicrobials mainly included β-lactamase inhibitors, Carbapenems, and Antifungals. CIP patients showed a clinically relevant but statistically non-significant increase in mortality (15.3% vs 8.3%, *p*=0.08) (**Figures 3**, **4**).

## 4 Discussion

In the real world, the immunochemotherapy is becoming increasingly common, and the incidence of pneumonia is also rising, often accompanied by CIP. Diagnosing CIP is challenging, especially when it coexists with pneumonia, which often requires the expertise of experienced clinicians and rigorous exclusionary diagnoses. Notably, prior predictive models seldom evaluated CIP risk in this combined therapy, and have typically excluded patients with pneumonia. Our research highlights that among these patients, factors including smoking history, COPD, chest CT scans of GGO or NSIP, elevated NLR, and low PaO_2_ should trigger heightened suspicion for CIP development.

This study indicates that patients with pleural effusion are less likely to develop CIP but are at a higher risk for pneumonia, which constitutes a significant finding. CIP is characterized by alveolar inflammation, with hyperactive T cells primarily affecting the pulmonary parenchyma, including alveoli and interstitium, over the pleura. The release of IFN-γ and TNF-α by CD8+ and Th1 cells triggers alveolar epithelial and endothelial apoptosis, promoting interstitial fibrosis with minimal pleural permeability increase, leading to infrequent pleural effusions (25, 31). In contrast, pneumonia causes effusions through toxin-induced vascular permeability and direct pleural invasion by pathogens like bacteria and tuberculosis, activating neutrophils and macrophages that release proteases and free radicals, disrupting the pleural barrier and causing exudation. Previous research has shown that pleural effusions are a rare AE associated with CIP. For instance, in the Check Mate 057 trial (32), a phase III study focusing on advanced squamous NSCLC, only 6% of patients in the nivolumab arm experienced pleural effusion, with none reported as treatment-related serious AE (33). Additionally, another study observed pleural effusion in 3 out of 9 CIP patients (34), with the most common radiological features being bilateral GGO and nodular shadows. Some scholars (35) have suggested that pleural effusion is an independent risk factor exclusively for severe CIP cases. Nonetheless, the correlation between pleural effusion and the occurrence of CIP versus pneumonia remains unsubstantiated by relevant research to date, necessitating further validation through large-scale studies in the future.

Notably, this study identified PaO_2_ and NLR as independent predictors for CIP, suggesting that PaO_2_ and NLR may serve as biomarkers for identifying CIP. Inflammatory cells and mediators within the tumor microenvironment are recognized to play pivotal roles in cancer progression and may contribute to this variability. Elevated NLR, an indicator of systemic inflammation, is known to portend poor prognosis across various cancers (36). In animal models (37), a 25% increase in NLR was observed in low-grade CIP compared to high-grade CIP. Among lung cancer patients, increased NLR levels correlated with CIP occurrence and severity (38). Matsukane et al. (39) analyzed NLR fluctuations in solid tumors and found a significant association between elevated NLR and the development of irAEs, particularly in CIP. Furthermore, elevated NLR levels at CIP diagnosis were linked to the occurrence of high-grade CIP, consistent with our findings. However, the delineation of NLR thresholds may vary depending on cancer type and location, potentially influenced by treatment, necessitating consideration of individual patient data in future studies. Our research found that decreased PaO_2_ is associated with the occurrence of CIP. In a previous analysis of five CIP patients (40), hypoxemia was observed in all cases. Additionally, a study (16) of 61 CIP patients reported that 19 patients (31.1%) experienced diffuse alveolar damage accompanied by hypoxemia, and these patients had significantly poorer prognosis. The main symptom of CIP patients is often dyspnea, which may be related to hypoxia. However, due to the small sample size, further validation by prospective studies is still needed.

The radiological manifestations of CIP are non-specific, often resembling infectious pneumonia or the progression of tumor infiltration or metastatic diseases (41). Recent studies (42–45) have shown that radiomics models based on data extracted from chest CT images demonstrate certain advantages in differentiating pulmonary irAEs from other pulmonary diseases. CIP is frequently associated with radiologic patterns such as organizing pneumonia (OP), NSIP, hypersensitivity pneumonitis (HP), obliterative bronchiolitis (OB), sarcoid-like reaction (SLR), or acute eosinophilic pneumonia (AEP). Previous studies have shown that the main chest CT findings are GGO (43.6%), followed by interstitial pneumonia (25.5%), OP(18.2%), and pneumonia not otherwise specified (PNOS) (12.7%) (18). Among the 51 CIP patients, 48 (94.1%) presented with GGO and/or patchy shadows, with air bronchograms in 14.6% of cases. Stride shadows were observed in 31.4% of patients, and consolidation was visible in 17.7% (30). This is consistent with the findings in the present study, where chest CT revealed GGO and NSIP, suggestive of possible of CIP.

Our study indicates that smoking history and underlying COPD are independent risk factors for CIP, consistent with previous research (23, 46, 47). This may be attributed to the persistent inflammatory state in lung tissues caused by COPD. Irreversible pulmonary parenchymal damage and chronic lung inflammation are risk factors for various drug-induced pneumonias, including CIP (48, 49).

In our study, no significant pathogen differences were found between subgroups, likely due to retrospective design, inconsistent detection, low traditional sputum culture positivity, some missing sputum results, and small sample size, which could all diminish the differences between groups. Larger, prospective studies with unified pathogen detection methods are needed to clarify differences. In the CIP group, the higher rate of antibiotic use could be attributed to the severity of the disease, suspected superimposed infections, as well as clinicians’ judgments and experiences. Notably, the rise in gamma globulin and albumin use in CIP patients likely results from severe infections after immunochemotherapy, aiming for immunomodulation. The study identified a significant difference in mechanical ventilation between CIP and No CIP patients, likely due to hypoxemia and dyspnea prevalent in CIP. The increased mortality in CIP patients is clinically relevant and may stem from the disease severity necessitating ventilation and potential diagnostic delays. Further research is needed to validate these findings.

The diagnosis of CIP remains challenging and primarily relies on medication exposure history, clinical symptoms, and radiological features. Zhou et al. (50) introduced the concept of Onco-Respirology, encompassing lung infections due to tumor-induced immunosuppression and antibiotic resistance, as well as tumor-treatment-related lung injuries caused by chemotherapy, ICIs, and radiotherapy. Thus, collaboration not only among respiratory physicians, oncologists, and thoracic radiologists but also with experts in basic medicine and pathology is imperative. Current CIP treatments (51) commonly encompass withdrawal of causative drugs and/or supportive care such as glucocorticoid use. Glucocorticoids remain the first-line therapy for CIP, and some patients have received empirical diagnoses based on their improvement after glucocorticoid therapy following ineffective antimicrobial treatment.

The nomogram developed in this study demonstrated excellent discriminative ability in the validation cohort (AUC=0.913). However, the wide confidence interval (0.826-0.999) warrants cautious interpretation. Given the small validation sample size (n=47), bootstrap resampling revealed potential moderate overestimation of the AUC (median 0.887). Nevertheless, decision curve analysis (DCA) showed improved net benefit over conventional strategies across clinically relevant risk thresholds (0.2-0.5), while the adjusted Brier score indicated acceptable overall prediction error. Future multicenter prospective studies are needed to optimize the model’s calibration performance.

Distinct from previous studies, this study addresses the clinical challenge of differentiating CIP from pneumonia, marked by high rates of misdiagnosis and missed diagnosis of CIP, and we selected pneumonia occurring after immunochemotherapy as the control group. We employed Random Forest machine learning to analyze predictive factors, optimizing the development-validation cohorts ratio for model efficacy. The developed nomogram demonstrates robust discriminatory capacity, offering a non-invasive and predictive management tool for these patients. especially beneficial for patients in primary hospitals and community centers. Utilizing multicenter data enhanced reliability and generalization of our findings. Our risk assessment nomogram may clinically benefit early diagnosis, minimizing reliance on subjective clinician judgment and facilitating timely interventions. For example, if the CIP prediction exceeds 60%, it suggests initiating glucocorticoid therapy immediately, reducing unnecessary antibiotic use. Regular monitoring of NLR and PaO_2_ can dynamically predict the risk of CIP, offering solid evidence for early identification by clinicians.

Our study has certain limitations. First, the small sample size and lack of external validation from independent cohorts. We optimized the development-validation cohorts ratio (e.g., 5:5, 7:3, 8:2) using random forest algorithms, ultimately selecting an 8:2 partition to balance performance and bias. Although random forests effectively manage complex interactions among predictors, their ‘black-box’ nature, which refers to the opacity of deep learning systems where the internal decision-making process is not easily interpretable, limits clinical interpretability. We addressed this by integrating logistic regression analysis through univariate and multivariate methods to identify key variables and used decision trees to ascertain each variable’s contribution to the model. Despite robust performance in our study, the small validation cohort and absence of external multicenter validation may introduce bias and overfitting. Second, as a retrospective study, the dataset was sourced from real-world data across multicenter, providing a representative reflection of clinical diagnostic practices in real-world settings. Despite these selection bias and unmeasured confounders are inherent limitations, though we maximized standardization across centers by having experienced radiologists, oncologists, and pulmonologists follow 2019 NCCN guidelines for patient inclusion and grouping. However, unrecorded comorbidities and confounders like N-terminal pro-B-type Natriuretic Peptide(NT-proBNP)and troponin, potential predictive factors not included, which could affect model performance. Thus, future larger-scale, prospective multicenter studies with independent validation are needed to confirm the nomogram’s clinical applicability.

## 5 Conclusion

This study developed and validated a nomogram, which provides a useful tool for clinicians to differentiate the occurrence of CIP among patients who develop pneumonia following immunochemotherapy. It demonstrates excellent discriminative power and predictive accuracy. Furthermore, the study compared the pathogens, management, and outcomes of patients with pneumonia and CIP. The proposed nomogram could enhance the risk assessment of CIP in this patient population.

## Data Availability

The raw data supporting the conclusions of this article will be made available by the authors, without undue reservation.
